# Aversive olfactory associative memory loses odor specificity over time

**DOI:** 10.1242/jeb.155317

**Published:** 2017-05-01

**Authors:** Christian König, Emmanuel Antwi-Adjei, Mathangi Ganesan, Kasyoka Kilonzo, Vignesh Viswanathan, Archana Durairaja, Anne Voigt, Ayse Yarali

**Affiliations:** 1 Research Group Molecular Systems Biology of Learning, Leibniz Institute for Neurobiology, 39118 Magdeburg, Germany; 2 Center for Behavioral Brain Sciences, 39118 Magdeburg, Germany

**Keywords:** Aversive associative memory, *Drosophila melanogaster*, Generalization, Long-term memory, Olfaction, Post-traumatic stress disorder, PTSD

## Abstract

Avoiding associatively learned predictors of danger is crucial for survival. Aversive memories can, however, become counter-adaptive when they are overly generalized to harmless cues and contexts. In a fruit fly odor–electric shock associative memory paradigm, we found that learned avoidance lost its specificity for the trained odor and became general to novel odors within a day of training. We discuss the possible neural circuit mechanisms of this effect and highlight the parallelism to over-generalization of learned fear behavior after an incubation period in rodents and humans, with due relevance for post-traumatic stress disorder.

## Abstract (precis)

**Highlighted Article:** Associative memories of noxious experiences can become detrimental if overly generalized; fruit fly aversive memories lose their specificity over time, mimicking the situation in rodents and humans.

## INTRODUCTION

Associatively learning the predictors of noxious events is useful for survival as it enables pre-emptive avoidance. Depending on the nature of the noxious experience, its memory can last a long time. In a variety of species and paradigms, long- versus short-term memories differ in molecular and cellular bases (Davis and Squire, 1984; Dudai, 2012; Kandel et al., 2014). We report that they also differ in their ‘information content’: in a fruit fly odor–electric shock associative memory paradigm, we observed a dramatic loss in the specificity of the learned avoidance for the trained odor, such that it became general to novel odors within a day of training. This change in the specificity of aversive memory (and of appetitive memory, as shown by Ichinose et al, 2015) with the passage of time is telling in terms of the fruit fly neural circuit mechanisms of memory consolidation and storage. Importantly, generalized fear and avoidance after an incubation period following a traumatic experience is a hallmark of post-traumatic stress disorder (PTSD) in humans and rodent models (Siegmund and Wotjak, 2006; Dunsmoor and Paz, 2015; Bergstrom, 2016; Jasnow et al., 2016). Although we are still far from an invertebrate model for PTSD, our present findings encourage mechanistic analyses of particular, well-defined symptoms of this disorder in the fruit fly – a simple, experimentally accessible system, well-suited for screening approaches.

## MATERIALS AND METHODS

**Fig. 1.**
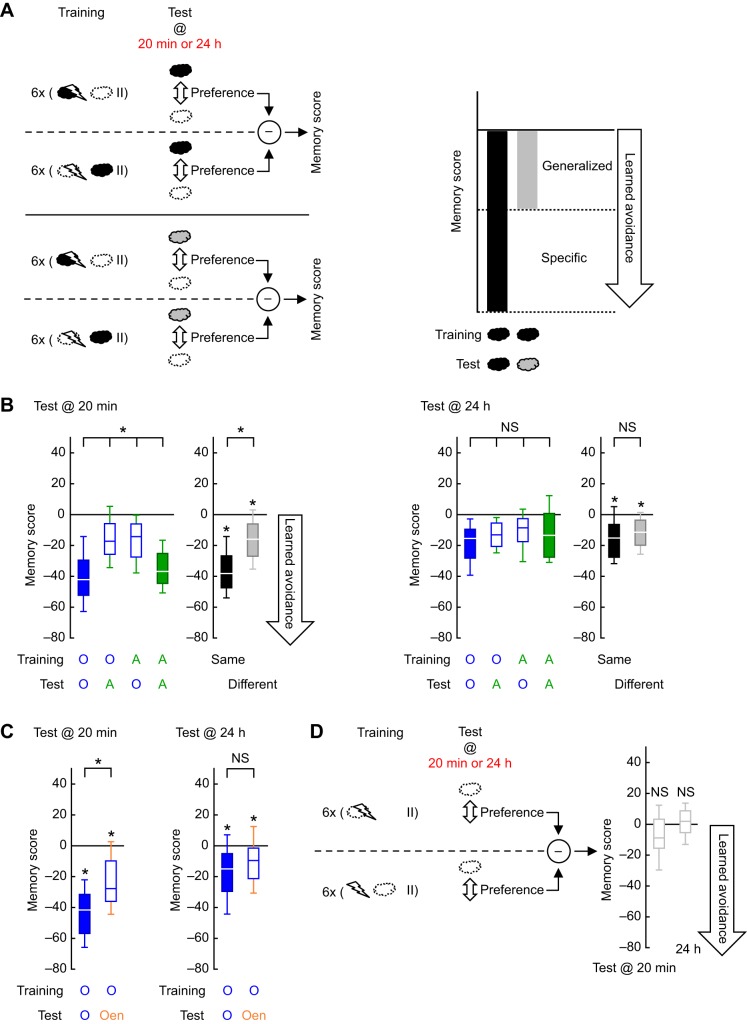
**Learned odor avoidance loses specificity over time.** (A) Top, left: two groups of flies were trained with either paired or unpaired presentations of an odor (black cloud; dashed cloud represents the solvent) and electric shock (lightning bolt). Then, 20 min or 24 h after repetitive training trials interspersed with pauses (pair of vertical lines), associative memory scores were calculated based on the difference between the odor preferences of the two groups, to reflect learned approach (>0) or avoidance (<0). Bottom, left: same as above, except a novel odor (gray cloud) was used for the test. Right: schematic explanation of how learned avoidance can be in part specific to the trained odor and in part generalized to a novel odor. Any difference between the black and gray bars would indicate specificity, while any difference of the gray bar from zero would indicate generalization. (B) Flies were trained with 3-octanol (O) or *n*-amylacetate (A) and tested with either the trained odor (conditions O–O and A–A) or the respective other odor (conditions O–A and A–O). Left: 20 min after training, memory scores significantly differed across the conditions O–O, O–A, A–O and A–A (KW-test: *H*=48.76, d.f.=3, *P*<0.0001, *N*=32, 40, 34, 46). Pooling the scores across the O–O and A–A as well as O–A and A–O conditions, which pair-wise did not differ (*U*-tests: O–O versus A–A, *U*=616.00, *P*=0.2248; O–A versus A–O, *U*=652.00, *P*=0.7655), we obtained two groups for which the training and test odors were either the same or different. In the ‘different’ group, memory scores were weaker than in the ‘same’ group (*U*-test: *U*=1002.00, *P*<0.0001). Significant learned avoidance was, however, detectable in each group (OSS-tests: *P*<0.0001 each). Right: 24 h after training, memory scores did not differ across the conditions O–O, O–A, A–O and A–A (KW-test: *H*=4.61, d.f.=3, *P*=0.2023, *N*=34, 40, 33, 47). Accordingly, ‘same’ and ‘different’ groups did not statistically differ and each reflected significant learned avoidance (*U*-test between ‘same’ and ‘different’: *U*=2546.50, *P*=0.1384; OSS-tests: *P*<0.0001 each). Thus, 20 min after training, learned avoidance was partially specific to the trained odor and partially generalized to a novel odor, whereas 24 h after training, no specificity was detected and generalization was full. In B–D: *P<0.05 in KW- or *U*-tests, *P<0.025 in OSS-tests; NS *P*>0.05 in KW- or *U*-tests, NS *P*>0.025 in OSS-tests. Box plots show the median, 25% and 75% and 10% and 90% quartiles as midline, box boundaries and whiskers, respectively. For the preference values underlying the memory scores, see Fig. S1A. (C) Flies were trained with O and tested with either O or 1-octen-3-ol (Oen). Left: 20 min after training, memory scores differed between O–O and O–Oen conditions (*U*-test: *U*=73.00, *P*=0.0059, *N*=20, 16) and significant learned avoidance was found in each case (OSS-tests: *P*<0.0001 for O–O and *P*=0.0213 for O–Oen). Right: 24 h after training, memory scores were the same under O–O and O–Oen conditions (*U*-test: *U*=156.50, *P*=0.2447, *N*=20, 20) and reflected significant learned avoidance in each case (OSS-tests: O–O, *P*=0.0026; O–Oen, *P*=0.0118). Thus, generalization was only partial 20 min after training, while 24 h after training, it was full. (D) Flies were trained as in A, but solvent and an empty odor container took the place of odor and solvent, respectively. The memory scores did not significantly differ from zero either 20 min or 24 h after training (OSS-tests: *P*=0.2478 and *P*=0.8506 for 20 min and 24 h, respectively; *N*=28, each).

**Fig. 2.**
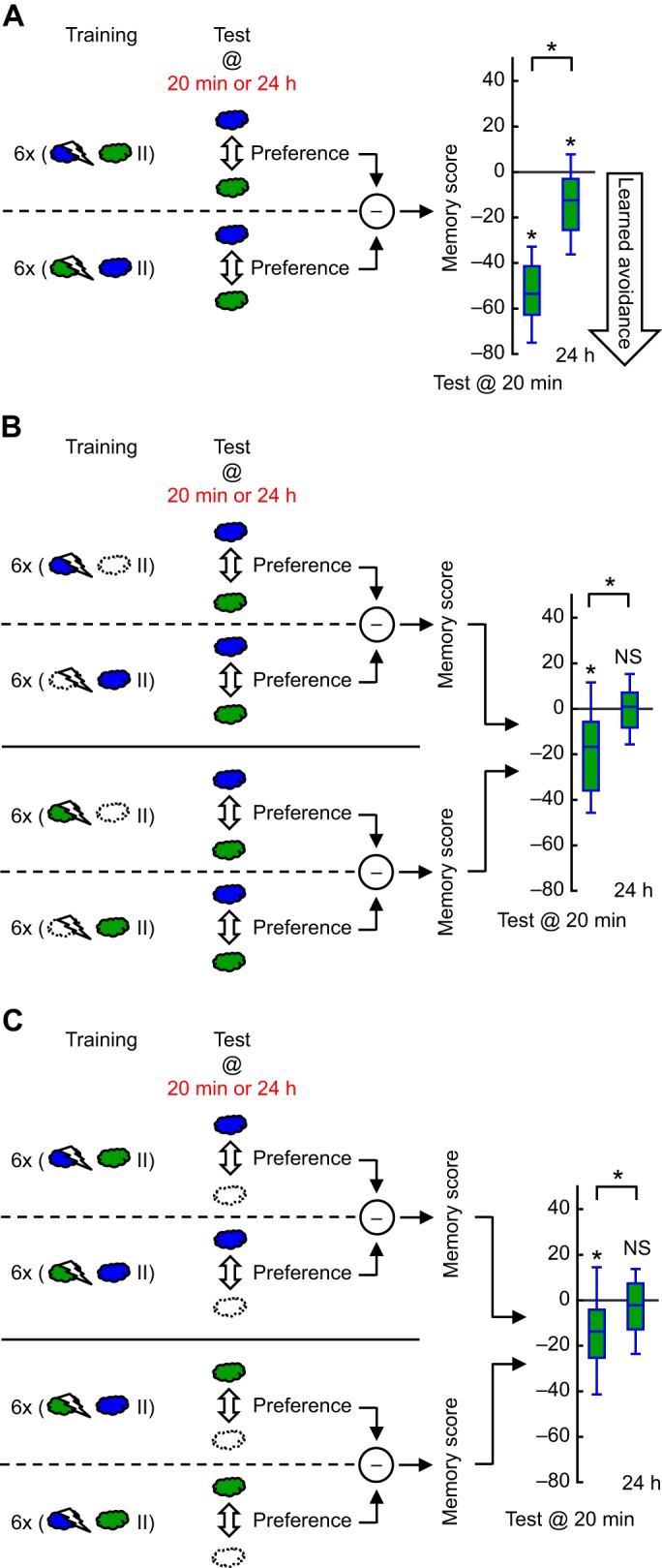
**Specificity of long-term learned avoidance is enhanced by discriminative training and discriminative testing.** (A) Two sub-groups of flies were trained with reversed roles for 3-octanol (O, blue cloud) and *n*-amylacetate (A, green cloud) as electric shock-paired and -unpaired odors. Other aspects of the experiment were as in Fig. 1A. Memory scores significantly deteriorated overnight (*U*-test: *U*=79.00, *P*<0.0001, *N*= 36, 36). Nevertheless, at both 20 min and 24 h after training, scores were significantly different from zero (OSS-tests: *P*<0.0001). Thus, even 24 h after discriminative training, learned avoidance retrieved in a discriminative test situation was at least partially odor specific. (B) Flies were trained and tested as in A, except one of the odors was replaced by odorless solvent during training. The scores did not depend on whether O or A was used during training (*U*-tests: 20 min after training with O versus A: *U*=96.00, *P*=0.3529, *N*=16, 15; 24 h after training with O versus A: *U*=112.00, *P*=0.7669, *N*=16, 15). Pooling across these conditions, we obtained the 20 min and 24 h groups, which are shown. Scores significantly deteriorated overnight (*U*-test: *U*=212.00, *P*=0.0002). In fact, significant learned avoidance was detected only at 20 min and not at 24 h (OSS-tests: *P*=0.0003 and *P*=0.7200 for 20 min and 24 h, respectively). Thus, 24 h after non-discriminative training, even a discriminative test situation could not retrieve any odor-specific learned avoidance. (C) Flies were trained and tested as in A, except one of the odors was replaced by odorless solvent at testing. As the results did not depend on whether O or A was used at testing (*U*-tests: test with O versus A 20 min after training: *U*=172.00, *P*=0.5903, *N*=24, 16; test with O versus A 24 h after training: *U*=191.00, *P*=0.9890, *N*=24, 16), we pooled across these conditions to obtain 20 min and 24 h groups. Scores significantly deteriorated overnight (*U*-test: *U*=532.00, *P*=0.0100); learned avoidance was detectable only in the 20 min group and not in the 24 h group (OSS-tests: *P*=0.0002 and *P*=0.6360 in 20 min and 24 h groups, respectively). Thus, 24 h after discriminative training, a non-discriminative test situation was not able to retrieve any odor-specific learned avoidance. * and NS are defined in Fig. 1B.

Canton-special wild-type *Drosophila melanogaster* were kept as mass cultures at 25°C, 60–70% relative humidity, under a 12 h:12 h light:dark cycle. Flies, 1–3 days old, of mixed sex were collected in fresh food vials and stored at 18°C, 60–70% relative humidity until they were 2–4 days old for the experiments, which were performed at 23–25°C, 70–80% relative humidity under white room-light. As odors, we used 3-octanol, *n*-amylacetate and 1-octen-3-ol (Merck, Darmstadt, Germany, CAS: 589-98-0, 628-63-7, 3391-86-4), diluted 100-fold (Figs 1 and 2; Fig. S2) or 30,000-fold (Fig. S1B) in paraffin oil (AppliChem, Darmstadt, Germany, CAS: 8042-47-5), presented in 14 mm diameter Teflon containers.

Flies were trained and tested in groups of ∼100. In Fig. 1, six training trials were spaced with pauses so as to support long-term memory formation (Tully et al., 1994). Each trial started by loading the flies into the setup (0:00 min); an odor, diluted in an odorless solvent (Fig. 1D) (see also Saumweber et al., 2011), was presented from 1:00 min for a period of 1 min; electric shock followed at 1:15 min as 12 pulses of 100 V, each pulse 1.2 s long and followed by the next pulse after 3.8 s pause. At 4:00 min, solvent was presented for 1 min. At 7:00 min, flies were removed from the setup into food vials and after a 14 min pause, a new training trial began. At the end of the sixth training trial, upon removal from the setup, flies were separated into two sub-groups. One sub-group was transferred 20 min later to the choice point of a test T-maze to distribute between odor versus solvent for 2 min. The other sub-group was left in the experimental room to be tested 24 h later. Preferences were calculated as:

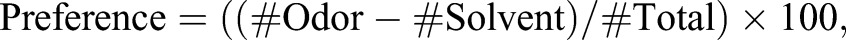


where # indicates the number of flies in the respective maze arm. For every group trained as odor–shock//solvent, another group received solvent–shock//odor training (// indicates a temporal gap). Based on the preferences of these two groups, we calculated a memory score as:

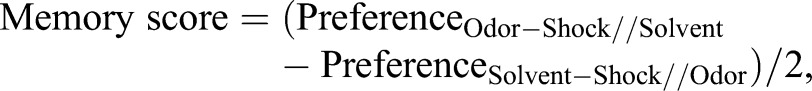
where the ‘Preference’ subscripts indicate the training regimen. Memory scores range from −100 to 100; positive values indicate learned approach, negative values indicate learned avoidance. Experiments were balanced in terms of which of the two groups was handled first and on which side of the setup the odor was presented during the test. In half of the experiments, the training was either odor–shock//solvent or solvent–shock//odor as explained above; in the other half of the experiments, the training started with solvent or odor presented at 1:00 min and continued from 4:00 min on with odor–shock or solvent–shock pairing.

Fig. S1B followed the protocol above, except either only one training trial was used or five trials were interspersed with 2 min pauses within the setup and the test started 6 min after the end of the last training trial. Fig. S2 used one training trial with 12 pulses of 50, 100 or 150 V shock with 3.8 s pauses or 24 pulses of 150 V shock with 1.3 s pauses, keeping all other parameters as in Fig. S1B. In Fig. 2, keeping all other parameters identical to Fig. 1, we replaced the solvent with a second odor during training and/or testing.

Memory scores were analyzed with Statistica version 11.0 (StatSoft, Hamburg, Germany) using Kruskal–Wallis tests (KW), Mann–Whitney *U*-tests (*U*), or one-sample sign tests (OSS). Bonferroni corrections limited the experiment-wide type 1 error rates at 0.05.

## RESULTS AND DISCUSSION

Using a fruit fly odor–electric shock associative memory paradigm, we asked whether the specificity of learned avoidance changes over time after training. Flies were given repetitive training trials spaced with pauses using a particular odor and were tested either shortly after or one day later with the trained odor or a novel one (Fig. 1A). In this design (Guerrieri et al., 2005; Niewalda et al., 2011), any deleterious effect of odor mismatch between training and test should reflect the specificity of learned avoidance, whereas memory scores remaining despite the mismatch should be due to generalization (Fig. 1A). When we applied this rationale to the odors 3-octanol (O) and *n*-amylacetate (A), memory scores measured 20 min after training suffered from a mismatch of odors between training and test, as reflected by the difference across the conditions training–test: O–O, O–A, A–O and A–A (Fig. 1B, left; statistical reports for all experiments are given in the figure legends). As the scores did not differ between either the conditions O–O and A–A, or the conditions O–A and A–O, we pooled the respective datasets to obtain two groups that encountered either the same or different odors in training versus testing. Although the scores were weaker in the ‘different’ group, learned avoidance was significant in each case. Thus, 20 min after training, learned avoidance was partially specific to the trained odor and partially generalized to a novel odor, fitting with the partial overlap between representations of these odors along the olfactory pathway (Niewalda et al., 2011). For those flies tested 24 h after training, however, a dramatically different picture emerged: overall weak but significant learned avoidance was found, which was not affected by a mismatch of odors between training and test (Fig. 1B, right). Thus 24 h after training, learned avoidance was not specific to the trained odor at all; instead, it was fully generalized to a novel odor. Using two other odors, 3-octanol and 1-octen-3-ol, with partially overlapping neural representations (Campbell et al., 2013; Barth et al., 2014), we corroborated the partial specificity of learned avoidance at 20 min after training, and the lack of specificity after 24 h (Fig. 1C).

Most studies of fly olfactory associative learning use discriminative training, where one of two consecutively presented odors is paired with electric shock, followed by a discriminative choice test between the two odors presented simultaneously in opposing arms of a T-maze (Fig. 2A). In Fig. 1, however, each experimental group was exposed to one odor – paired or unpaired with shock – during training, and a single odor – trained or novel – was presented at test. In such a non-discriminative paradigm, long-term learned avoidance was completely generalized to an odor different from the trained one, as far as the odors O, A and Oen were concerned (Fig. 1B,C). This led us to a straightforward prediction: using the same odors, 24 h after discriminative training, memory scores based on a discriminative test should be zero, as equal learned avoidance would be applicable to the two odors regardless of whether they were paired with shock or not. We tested this prediction using a discriminative training and test design, keeping all other parameters the same as in Fig. 1B. The scores significantly deteriorated from 20 min to 24 h after training (Fig. 2A), which may reflect, in addition to a decay of memory strength, a loss of specificity. Importantly, however, 24 h after training, scores were still significantly different from zero (Fig. 2A), indicating that learned avoidance remained at least partially specific to the respective shock-paired odors. This boost of odor specificity for long-term learned avoidance required both the training and the test to be discriminative: using a discriminative situation in only one of these phases did not give significant memory scores at 24 h (Fig. 2B,C).

Our findings can be summarized as follows. (i) Learned avoidance after associative odor–electric shock training lost its specificity for the trained odor with the passage of time, as previously shown for odor–food reward memory in adult flies (Ichinose et al., 2015). (ii) The specificity of long-term learned avoidance was enhanced when training and testing explicitly promoted and required discrimination between two odors, paralleling the situation with respect to short-term aversive and appetitive memories in adult and larval *Drosophila*, respectively (Barth et al., 2014; Mishra et al., 2010). (iii) Other parameters of training, i.e. the number of repetitions or reinforcement strength, did not affect the specificity of learned avoidance within the range we looked at (Figs S1B, S2). The lack of effect of reinforcement strength may be unsurprising, given that in honey bees even reversing reinforcement valence did not influence memory specificity (Bos et al., 2014).

Although avoiding associatively learned predictors of danger is crucial for survival, aversive memories can become detrimental when overly generalized to harmless cues and contexts, as happens in PTSD following a period of incubation (Siegmund and Wotjak, 2006; Dunsmoor and Paz, 2015; Bergstrom, 2016; Jasnow et al., 2016; Andreatta et al., 2016). Mimicking this situation in humans, rodent learned fear behavior becomes more general over time following contextual or cued aversive learning (Wiltgen and Silva, 2007; Pamplona et al., 2011; Jasnow et al., 2016). Here, we made a corresponding observation in the fruit fly (Fig. 1).

Obviously, animal models of PTSD must mimic a variety of symptoms before they gain face validity (Siegmund and Wotjak, 2006; Yehuda and LeDoux, 2007; Pitman et al., 2012; Parsons and Ressler, 2013; Dunsmoor and Paz, 2015; Bergstrom, 2016; Jasnow et al., 2016). It may not be possible to address all of these symptoms in non-humans; however, some symptoms may be operationally mimicked even in the fruit fly, as perhaps is the case for over-generalization (Fig. 1). In this sense, it seems also relevant that: (i) flies form not only aversive memories about cues preceding an electric shock but also relief memories about cues that follow a shock (Tanimoto et al., 2004; Yarali et al., 2008; Vogt et al., 2015). The net effect of a noxious experience is thus shaped by a mnemonic opponency in the fly as in rodents and humans (Solomon and Corbit, 1974; Gerber et al., 2014). (ii) Flies' learned avoidance after odor–electric shock training deteriorates upon repeated encounters with the odor in the absence of shock (Schwaerzel et al., 2002), corresponding to extinction, a protective mechanism against PTSD. (iii) Echoing the inter-individual variability of behavioral consequences of trauma in humans and rodents, the strength of memories about an experience with electric shock varies across inbred fly strains (Appel et al., 2016). Such natural genetic variation among flies can be studied with respect to generalization or extinction too. (iv) Fly sleep, eating, courtship and aggression paradigms are available for exploring the richness of behavioral effects of a noxious experience, given that in humans and rodents, these extend beyond avoidance. Thus, the fruit fly, despite its genomic and neuronal simplicity, may potentially provide the necessary behavioral complexity for tackling particular, well-defined symptoms of PTSD using the advantage of a matchless transgenic toolbox and screening approaches that are less feasible in other models.

The difference of specificity between short- and long-term aversive memories should have its basis in the underlying engrams, which are well studied in the fruit fly (Heisenberg, 2003; Gerber et al., 2004; Owald and Waddell, 2015). During odor–electric shock training, the odor is signaled through a side-branch of the olfactory pathway to the mushroom bodies, where odors are coded sparsely across the Kenyon cells (KCs). A shock-induced reinforcement signal is also delivered to the KCs via dopaminergic neurons. In those KCs that respond to the trained odor, the coincidence of these two signals triggers the molecular events leading to a modification of the output synapses to downstream mushroom body output neurons, changing the net behavior to the particular odor in favor of avoidance. This behavioral change is generalized to other odors to the extent that their representations overlap with the representation of the trained odor along the olfactory pathway (Niewalda et al., 2011; Campbell et al., 2013; Barth et al., 2014). Interestingly, at the level of the KCs, discriminative olfactory aversive training renders the representations of the respective odors more dissimilar, providing a neural correlate for the enhanced specificity of learned avoidance in the short term (Barth et al., 2014) and possibly also the long term (Fig. 2). Importantly, KCs come in three classes, each interacting with particular dopaminergic and mushroom body output neurons at distinct regions along their axons, resulting in a compartmental organization (Aso et al., 2014a). Short- versus long-term memories rely on different mushroom body compartments (Pascual and Préat, 2001; Blum et al., 2009; Cervantes-Sandoval et al., 2013; Aso et al., 2014b; Aso and Rubin, 2016). It is thus conceivable that the respective engrams are formed in parallel at distinct cellular sites. The difference in specificity between them (Fig. 1) could then be due to a difference in odor coding between the respective KC classes. However, for a stable memory to be formed, the activity of particular neurons in the mushroom body-centered circuit is also critical during the resting periods between training trials or between training and testing (Krashes et al., 2007; Plaçais et al., 2012; Ichinose et al., 2015). Therefore, it is also possible that the engram loses its odor specificity in a systems consolidation-like process (Dudai, 2012; Ichinose et al., 2015). Critical empirical tests for the scenarios outlined above would include a systematic comparison of odor coding across different KC classes (Murthy et al., 2008; Turner et al., 2008) and characterization of odor specificity of memories that are artificially induced in various mushroom body compartments (Aso and Rubin, 2016). A detailed account of how the passage of time changes the specificity of aversive memory in the relatively simple brain of the fruit fly may aid understanding of over-generalization of learned fear in rodents and humans.
